# Arthroscopic approach does not yield better results than open surgery after subscapularis repair: a systematic review

**DOI:** 10.1007/s00167-023-07403-1

**Published:** 2023-04-01

**Authors:** Vito Gaetano Rinaldi, Matteo La Verde, Federico Coliva, Eugenio Cammisa, Giada Lullini, Silvio Caravelli, Massimiliano Mosca, Stefano Zaffagnini, Giulio Maria Marcheggiani Muccioli

**Affiliations:** 1 II Clinica Ortopedica e Traumatologica, IRCCS Istituto Ortopedico Rizzoli, Via di Barbiano, 1/10 – c/o Lab Biomeccanica ed Innovazione Tecnologica, 40136 Bologna, Italy; 2 DIBINEM, University of Bologna, Bologna, Italy; 3 Unità Ortopedica Ospedale di Imola, Imola, Italy; 4 UOC Medicina Riabilitativa e Neuroriabilitazione, IRCCS Istituto delle Scienze Neurologiche, Bologna, Italy

**Keywords:** Subscapularis tears, Arthroscopy, Arthroscopic repair, Mini-open repair, Rotator cuff, Sport medicine, Shoulder surgery, Shoulder trauma

## Abstract

**Purpose:**

This study aimed to compare the long-term outcomes of arthroscopic versus mini-open repair in patients with isolated subscapularis tendon tears.

**Methods:**

Google Scholar, PubMed, and Embase databases were searched for studies evaluating isolated subscapularis tears subsequently treated by arthroscopic or mini-open repair. The inclusion criteria were clinical studies reporting isolated subscapularis lesions treated by arthroscopic or mini-open repair, a minimum follow-up of 12 months, and clinical and functional outcomes reported in the study results. Articles not reporting functional outcomes or studies that reported results for anterosuperior rotator cuff tears without a separate analysis of subscapularis tendon tears were excluded. Studies older than 20 years and studies with a minimum follow-up of less than 12 months were also excluded.

**Results:**

A total of 12 studies met the inclusion criteria; 8 papers were included in the arthroscopic repair group, and 6 were included in the mini-open repair group (2 studies reported results for both techniques). The mean age reported was 49.3 years, and 85.1% of patients were male. The dominant limb was involved in 77.6% of the patients, and a traumatic onset of symptoms was verified in 76.3%. The mean time to surgery was 9.6 months. The Constant–Murley score showed positive results for the arthroscopic and mini-open groups, with mean postoperative values of 84.6 and 82.1, respectively. Promising results were also observed for pain, with a mean of 13.2 (out of 15) points for the arthroscopic group and 11.7 for the mini-open group. The long head of the biceps was involved in 78% of the patients, and LHB tenodesis or tenotomy were the most common concomitant procedures performed.

**Conclusions:**

There was no significant difference in clinical and functional outcomes between open and arthroscopic repair. Moreover, the same complication rates were reported in both treatments, but arthroscopic repair led to less postoperative pain.

**Level of evidence:**

IV.

## Introduction

The subscapularis (SSC) muscle is the rotator cuff's strongest muscle, with a bulk mass superior to that of all 3 other rotator cuff (RC) muscles combined, allowing internal humeral rotation, preventing anterior dislocation of the humeral head from the glenoid fossa, and providing long head biceps (LHB) stability [39].

Despite its importance, the subscapularis has been labelled as the forgotten rotator cuff tendon because of the meagreness of the literature surrounding its repair compared with that describing the supraspinatus and infraspinatus repair techniques. This is likely because subscapularis tears are rare and technically challenging to repair compared to either supraspinatus or infraspinatus tears. Only 3–4% of rotator cuff tears involve the subscapularis [1], and isolated subscapularis tears are even rarer [38].

However, in recent years, a better understanding of anatomy and biomechanics combined with improved imaging technology and the increased use of arthroscopy has led to a higher rate of subscapularis tear diagnoses and repairs. Furthermore, many classifications for subscapularis tendon tears have been proposed to date [23, 24, 30, 37, 40]. Subscapularis tears can be isolated, part of an anterosuperior rotator cuff tear, or a continuum of large and massive rotator cuff involvement [26, 34].

As shown by several authors, regardless of the type of damage, its repair provides stability and better biomechanical function to the glenohumeral joint, especially in young and athletic patients [4–7, 12, 14, 17, 28, 29]. Therefore, various surgical techniques, including open, mini-open, and all-arthroscopic repair techniques, have been previously proposed by surgeons for isolated subscapularis tendon tears. In recent years, many authors have debated whether an arthroscopic or mini-open repair technique is preferable [5, 12, 21].

The present study aims to systematically review and compare mini-open and arthroscopic surgical repair techniques for isolated subscapularis tears and present the associated results. The necessity to present this paper stems from the need for a recent study collecting, updating, and comparing data on isolated subscapularis repair.

## Materials and methods

### Literature search

The literature search was performed in PubMed (MEDLINE and Embase) and Google Scholar on April 1st, 2022, by three researchers. The string used for the search was “(subscapularis[Title/Abstract]) AND (arthroscopic[Title/Abstract])) AND (mini-open[Title/Abstract])) OR (mini open[Title/Abstract])) OR (open[Title/Abstract])) AND (repair[Title/Abstract])) AND (tendon[Title/Abstract])) AND (rotator cuff[Title/Abstract])”.

All relevant studies between 1990 and 2022 were identified in accordance with the Preferred Reporting Items for Systematic review and Meta-Analysis (PRISMA) guidelines (Table 1) [33]. The first selection of studies was made by examining the article titles. The papers that passed the first selection were subjected to careful analysis of the abstract. The authors also evaluated the bibliographies of the included articles to search for further studies that were added later to our review if they met all the inclusion and exclusion criteria.

**Table 1:**
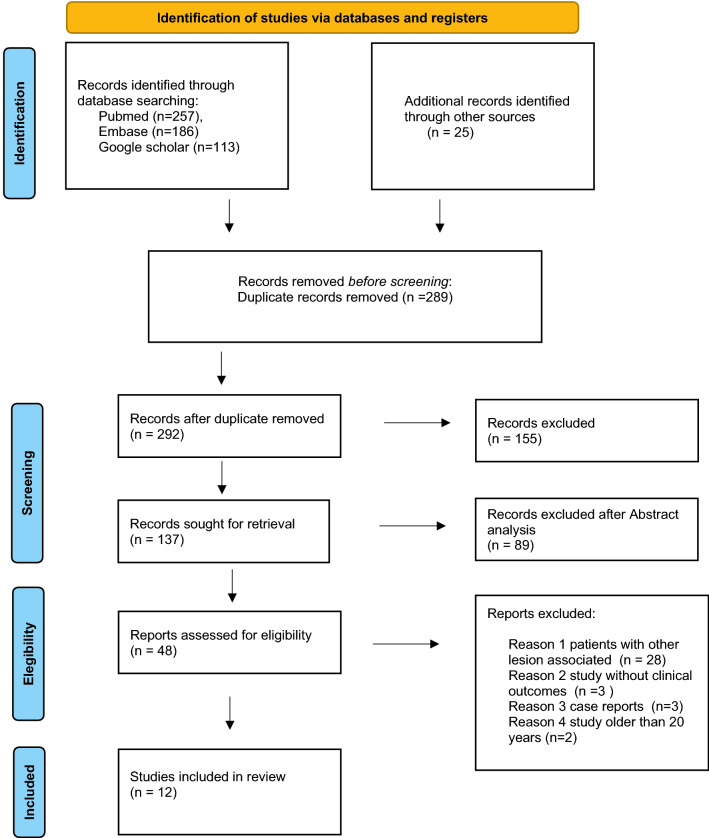
Prisma flowchart

All the selected articles adhered to the Population, Intervention, Comparison, and Outcomes (PICOS) criteria for systematic reviews [16].

### Eligibility criteria

The inclusion criteria were as follows: (1) the study reported isolated lesions of the subscapularis, (2) the patients with subscapularis tendon lesions were treated with an open repair or arthroscopy repair, (3) the article reported the functional outcomes following each treatment, and (4) a minimal clinical follow-up of 12 months was needed.

The exclusion criteria were as follows: (1) articles not reporting functional outcomes, (2) outcome studies that reported results for anterosuperior rotator cuff tears or combined rotator cuff tears without a separate analysis for subscapularis tendon tear, (3) case reports, (4) studies older than 20 years, and (5) studies with a minimum follow-up of less than 12 months.

### Data extraction

The data were extrapolated from the selected documents using a standardized data collection form. Study data collected included the year of publication, type of clinical study, level of evidence (I–IV), type of repair (open vs. arthroscopic), study period, inclusion/exclusion criteria, number of patients enrolled, number of patients available for follow-up, age, length of follow-up, proportion of dominant extremities involved, and different clinical outcomes reported by each study.

Functional outcomes that were of interest included the University of California, Los Angeles (UCLA) outcome score, Constant–Murley (C–M) outcome score, Pennsylvania Shoulder Score, American Shoulder and Elbow Surgeons (ASES) outcome score, Simple Shoulder Test (SST), visual analogue scale (VAS) for pain, and overall patient satisfaction rates. Finally, the presence of bias was determined and analysed for each eligible study.

### Quality assessment

Two authors assessed the quality and rigor of the included studies using the methodological index for non-randomized studies (MINORS). The ideal global score is 16 for noncomparative studies and 24 for comparative studies. The items were scored 0 if not reported, 1 if reported but inadequate, and 2 if reported and adequate. Consensus was reached by the two reviewers (FC and MLV) when there was no difference in opinion on an item. If no consensus was reached, the independent opinion of a third reviewer was decisive (VGR). The individual scores are reported in Table 2.

**Table 2:** Minors criteria

	Edwards et al. (2005) [13]	Fuchs et al. (2006) [17]	Kreuz et al. (2005) [22]	Lafosse et al. (2007) [23]	Bennet (2003) [7]	Novè-Josserand et al. (2012) [31]	Grueninger et al. (2014)	Heikenfeld et al. (2012) [20]	Lanz et al. (2013)	Novè-Josserand et al. (2016) [32]	Bartl et al. (2011) [5]	Bartl et al. (2011) [5]
A clearly stated aim	2	2	2	2	2	2	2	2	2	2	2	2
Inclusion of consecutive patients	0	2	2	2	2	2	2	2	2	2	2	2
Prospective collection of data	2	2	2	2	2	2	2	2	2	2	2	2
Endpoints appropriate to the aim of the study	2	2	2	2	2	2	2	2	2	2	2	2
Unbiased assessment of the study endpoint	0	0	1	0	0	0	0	0	0	0	0	0
Follow-up period appropriate to the aim of the study	2	2	2	2	2	2	2	2	2	2	2	2
Loss to follow up less than 5%	0	2	2	2	2	2	2	2	0	0	2	2
Prospective calculation of the study size	2	2	2	2	2	2	2	2	2	2	2	2
An adequate control group	0	0	0	0	0	2	0	0	0	0	0	0
Contemporary groups	0	0	0	0	0	2	0	0	0	0	0	0
Baseline equivalence of groups	0	0	0	0	0	2	0	0	0	0	0	0
Adequate statistical analyses	0	0	0	0	0	2	0	0	0	0	0	0
	10	14	15	14	14	22	14	14	12	12	14	14

The study quality of the information reported in the included manuscripts was based on the Strengthening the Reporting of Observational studies in Epidemiology (STROBE) checklist criteria, which is a reliable quality rating tool for observational studies [2, 15]. Each criterion was scored as “yes”, “no”, or “not applicable (NA)”. A criterion was scored as “yes” if it was applicable and met in the study, “no” if it was applicable but not met, and “NA” if it was not relevant to the study. The scores obtained were compared among the reviewers to assess the importance and validity of each individual study. The number of criteria scored as “yes” divided by the number of applicable criteria per manuscript yielded a percentage of the applicable STROBE criteria [10].

All articles examined had a STROBE percentage score greater than 90%. This highlights how, even though the studies were conducted in different years, there was a strong focus on following the correct formation of a scientific article by each author.

### Statistical analysis

The results were summarized using descriptive statistics for continuous variables and frequencies and percentages for categorical variables. All statistical analyses were performed in Microsoft Excel, 2016 version (Microsoft Corporation, Redmond, WA, USA).

## Results

### Study population and demographics

The first search produced 581 studies using PubMed, Embase, and Google Scholar as research browsers; 289 duplicates were removed, leaving 292 studies. The titles and abstracts were screened to remove 155 and 89 studies, respectively, leaving 48 studies included in our final evaluation based on the inclusion/exclusion criteria described in our PRISMA flowchart (Table 1). Finally, 12 papers were included in our review.

Demographic data for the isolated subscapularis lesion group were not reported in 2 out of the 12 studies selected. One study had isolated demographic aspects for both open repair and arthroscopic repair, so we reported the two groups separately in Table 2 [31]. The population characteristics we considered, as shown in Table 3, were age, sex, average follow-up time, dominance of the limb involved, onset on a traumatic basis, and time elapsed between the injury and surgery. All the mean values shown were adjusted by the size of the population of each study.

**Table 3:** Study characteristics and patient demographics

	LOE	S. D	STROBE (%)	OSR	ASR	Age (range)	Males (%)	Mean F-U (range)	Dominant side (%)	Traumatic Onset (%)	Mean T.t.S. (range)
Edwards et al. (2005) [13]	IV	RCS	90.9	84		53 (23–77)	70 (83.3%)	45 (24–132)	65 (77.4%)	57 (67.9%)	12.5 (0–108)
Fuchs et al. (2006) [17]	IV	RCS	90.9	10							
Kreuz et al. (2005) [22]	IV	RCS	90.9	16		46 (27–64)	14 (87.5%)	36 (28–48)	15 (93.75%)	16 (100%)	3 (0.25–8)
Lafosse et al. (2007) [23]	IV	PCS	95.4		17	47 (29–59)	13 (76.47%)	29 (24–39)	16 (94.11%)	13 (76.47%)	24 (3–44)
Bennett (2003) [7]	IV	PCS	90.9		8	56 (32–76)	5 (62.5%)	36 (24–48)	8 (100%)		
Novè-Josserand et al. (2016) [32]	IV	RCS	95.4	16	24	52.4 (43–61.8)	36 (90%)	42 (25–69.6)		24 (60%)	
Grueninger et al. (2014)	IV	PCS	95.4		11	45 (32–65)	9 (81.8%)	12	8 (72.7%)	10 (90,9%)	3.7 (0.3–13.3)
Heikenfeld et al. (2012) [20]	IV	PCS	90.9		20	42 (31–56)	18 (90%)	24	16 (80%)	19 (95%)	6.7 (0–18)
Lanz et al. (2013)	IV	PCS	90.9		7						
Novè Josserand et al. (2012) [31]	IV	RCS	95.4	13		49.5 (22–62)	13 (100%)	47.8 (36–57)	9 (69%)	8 (62%)	
Novè Josserand et al. (2012) [31]	IV	RCS	95.4		22	54.7 (46–74)	20 (90.9%)	35.7 (25–49)	14 (64%)	13 (59%)	
Bartl et al. (2011) [5]	IV	PCS	95.4	30		43.1 (15–64)	26 (86.7%)	46 (25–72)	22 (73.3%)	30 (100%)	4.1 (0,2–15)
Bartl et al. (2011) [5]	IV	PCS	90.9		21	43.7 (18–61)	16 (76.2%)	27	15 (71%)	19 (90.5%)	5.8 (0.2–14)
Mean adjusted by population						49.4	85.1	38.2	77.7	76.3	9.7

*LOE* Level of evidence, *SD* Study design, *RCS* retrospective case series, *PCS* prospective case series, *OSR* open surgery repair number of patients, *ASR* arthroscopic surgery repair number of patients, *Age* reported in years, *F-U* Follow-up expressed in months, *T.t.S.* Time to Surgery expressed in days

The mean age reported in the studies was 49.4 years, ranging from 15 to 77 years. In all studies, the population had a majority of males, with a mean percentage of men of 85.1% (range 62.5–100%). In all studies, a predominance of involvement of the dominant limb and a traumatic onset of the injury were reported: the dominant side was affected in 77.6% of the cases (range 64–100%), and a traumatic cause was found in 76.3% (range 59–100%). Every study had a minimum follow-up of 2 years, except one that reported results at 12 months after surgery. Only seven studies reported the average time elapsed between symptom onset and surgery, ranging from 0 to 108 months.

### Clinical outcomes

The clinical results are presented in Table 4. At the final data evaluation, 8 studies were included in the arthroscopic repair group for a total of 128 shoulders examined, whereas 6 were included in the open repair group for a total of 169 shoulders. One study compared arthroscopic and open repair techniques, so the reported data were considered in both groups [31]; one study divided patients into a complete tendon rupture group and a partial tendon rupture group, so we reported the data separately [22].

**Table 4:** Clinical score and tests

	Patients	CMS		2nd Score			Pain scale		B-P T		L-O T	
		Preop	Postop		Preop	Postop	Preop	Postop	Preop	Postop	Preop	Postop
Arthroscopic repair												
Lafosse et al. (2007) [23]	17	52	84.9	UCLA	16.2	32.1	5.9	13.5	9	4	11	4
Bennett (2003) [7]	8	43.25	74.17	ASES	16.11	74.44	9 (1.5)	2 (12)				
Novè-Josserand et al. (2016) [32]	24	65.6	85.3						14 P, 4 A	5 (out of 36)		7 (out of 36)
Grueninger et al. (2014)	10	43.5	89.3				4.6	13.2	7	0	4	0
Heikenfeld et al. (2012) [20]	19	41.3	81	UCLA	16.5	32.5			16	2	13 (out of 15)	4
Novè Josserand et al. (2012) [31]	22	66.4	85.2				5.05	13.09	12 P, 4 A	3 P, 7 A		5 P, 8 A
Lanz et al. (2013)	7	46.2	77.3	UCLA	14.2	30						
Bartl et al. (2011) [5]	21	50.3	82.4	SST	6.3	11.2	4.4	13.3	19 P	5	16 P (out of 16)	1 P, 1 A
	128											
Mean values		54.3	84.6				5	13.3				
Mini-open repair												
Edwards et al. (2005) [13]	84	55	79.5				4.6	11.2	76	15	60	17
Fuchs et al. (2006) [17]	10	51.8	72.9				5	11.8				
Kreuz et al. (2005) [22]	9	38.7	89.3						9		9	0
Kreuz et al. (2005) [22]	7	50.7	87.9						1		4	0
Novè-Josserand et al. (2016) [32]	16	68.6	90.5						7 P, 5 A	5 (out of 36)		7 (out of 36)
Novè Josserand et al. (2012) [31]	13	67.4	88.4				5.3	13.2	6 P, 4 A	2 P, 3 A		2 P. 5 A
Bartl et al. (2011) [5]	30	51.3	82.2	SST	5.8	11.2	4.2	12.6	27	6 A	17 (out of 21)	3 (out of 28)
	169											
Mean values		55.4	82.2				4.6	11.7				

*Patients* population reported in paper, *CMS* Constant–Murley Score, *2nd score* 2nd score used in paper if present, *UCLA* UCLA shoulder score, *ASES* American Shoulder and Elbow Surgeons standardized shoulder assessment form, *SST* Simple Shoulder Test, *Pain scale* evaluated with a Visual Analog Scale included in the CMS evaluation with a maximum of 15 points, *B-P T* Belly-Press test, *L-O T* Lift-Off test, *Preop* preoperative, *Postop* postoperative, *P* frankly positive test, *A* asymmetrical if compared to contralateral, *(out of x)* variation of the total population considered either for how data were reported or for inability to perform test, *Mean Values* mean value adjusted by population size of each paper

As shown in Table 4, every paper reported the preoperative and postoperative Constant–Murley Score (CMS) [9]. Three studies also used the UCLA shoulder score [3], two used the Simple Shoulder Test (SST [18]), and one also used the ASES score [27]. Out of the 15 studies included in this review, only 8 reported pain values before and after surgery.

The mean preoperative value of the Constant score was 54.2 in the arthroscopic group and 55.3 in the open group; after surgery, the mean values increased to 84.63 in the arthroscopic repair group and 82.18 in the open repair group. As expected, the Constant score values were significantly different after arthroscopic and open surgery compared with the respective preoperative values. Nevertheless, no statistically significant difference was found between the two techniques.

Nine reported studies used the Belly Press Test (BPT) and the Lift-Off Test (LOT) for pre- and postoperative clinical evaluation and diagnosis. As shown in Table 4, LOT execution in the preoperative assessment was not possible in any of the patients examined because of the pain generated by this manoeuvre. The results of these tests were reported as either positive or asymmetrical (A) if the strength was inferior to the contralateral side but the test was not definitively positive. The study published by Novè-Josserand et al. in 2016 did not report the results of the clinical tests divided into the arthroscopic and mini-open groups, but data for the total population of the study were reported [32].

In Table 5, we reported the data about surgical technique, imaging, and the involvement of LHB and concomitant procedures.

**Table 5:** Patient characteristics and surgical technique

	Patients	Imaging		Goutallier	Rep Tech	LHB status	Conc Proc
		Preop	Postop				
Arthroscopic repair							
Lafosse et al. (2007) [23]	17	CTA	CTA	15 0-I, 2 II, 0 III	Anchors	8 N, 7 PR, 2 R,	9 LHB td
Bennett (2003) [7]	8				Anchors		4 coracoplasty
Novè-Josserand et al. (2016) [32]	24	13 MRI, 22 CTA, 5 MRI + CTA	35 MRI, 5 CTA	19 0-I, 5 II, 0 III	Anchors	6 N,18 INS	19 LHB td, 3 LHB tt
Grueninger et al. (2014)	10	11 MRA	10MRA		Anchors	10 INS, 2 PT	
Heikenfeld et al. (2012) [20]	19	20 MRI	19 MRI	17 0-I, 3 II, 0 III	Anchors	6 N, 1 R, 12 PT/INS	12 LHB td
Novè Josserand et al. (2012) [31]	22	22 MRI /CTA	22 MRI /CTA	19 0-I, 3 II, 0 III	Anchors		
Lanz et al. (2013)	7	7 MRI/CTA	7 MRI/CTA		Anchors		
Bartl et al. (2011) [5]	21	21 MRI	21 MRI		Anchors	3 N, 7 PR, 2 R, 9 INS	9 LHB td, 1 LHB tt, 2 rec
	128						
Mini-open repair							
Edwards et al. (2005) [13]	84	72 CTA, 84 RX	84 RX, 5 CTA	55 0-I, 13 II, 4 III-IV	Anchors, Staples, TO		48 td, 13 tt, 4 rec, 11 excission of distal clavicle
Fuchs et al. (2006) [17]	10	10 MRI	10 MRI		10 TO over plate		
Kreuz et al. (2005) [22]	9	16 CTA + MRI or US			7 Anchors, 7 TO, 2 T-T suture		
Kreuz et al. (2005) [22]	7						
Novè-Josserand et al. (2016) [32]	16	13 MRI, 22 CTA, 5 MRI + CTA	35 MRI, 5 CTA	12 0-I, 3 II, 0 III	Anchors	4 N, 12 INS	13 LHB td
Novè Josserand et al. (2012) [31]	13	MRI/CTA	MRI/CTA	11 0-I, 2 II, 0 III	Anchors		
Bartl et al. (2011) [5]	30	30 MRI o US	28 MRI o US	9 0-I, 11 II, 10 III	Anchors	3 N, 3 R, 24 INS	17 td, 3 tt, 7 rec
	169						

*Imaging* exams used for diagnosis and postoperative evaluation, *Goutallier* preoperative Goutallier grade of subscapularis fatty infiltration, *Rep Tech* repair technique used in the study, *LHB* Long Head Biceps, *Conc Proc* Concomitant procedures during surgery, *Patients* number of patients reported, *Preop* preoperative, *Postop* postoperative, *CTA* computed tomography arthrography, *MRI* magnetic resonance imaging, *MRA* magnetic resonance arthrography, *RX* radiography, *US* ultrasonography, *0-I/II/III* Goutallier grade, *TO* transosseous, *TO over plate* transosseous tied over a plate, *T-T* tendon to tendon suture, *N* normal, *PR* partial rupture, *R* rupture, *INS* instable, *LHB td* long head biceps tenodesis, *LHB tt* tenotomy, *rec* recentered

The most commonly used surgical technique used anchors to reinsert the torn tendon on the medial aspect of the bicipital groove; only 3 papers out of 12 reported other techniques, such as transosseous suture, tendon-to-tendon suture, staples, and a thin cortical plate over which the tendon was sutured [13, 17, 22]. All 3 studies were included in the mini-open surgery group.

Only one study did not report the imaging system used to diagnose SSC tendon rupture. Ten studies out of the eleven remaining used either MRI, MRA, or CTA to analyse every patient, while Edwards et al. [13] used CTA in 72 patients out of 84, and the remaining 12 underwent normal RX examination. The Sugaya classification was used in two studies to confirm the diagnosis of SSC tendon rupture [31, 32, 35].

Six studies reported the preoperative Goutallier fatty infiltration classification [19], and only two studies treated patients with grade III or IV [5, 13].

Pain was evaluated using a 15-point visual analogue scale (VAS) included in the CMS [9] in 7 studies, while one study used a 10-point VAS [11], and another study used a nominal pain scale. In the arthroscopic group, the four studies using the 15-point pain scale reported a mean preoperative value of 5 and a mean postoperative value of 13.3. On the other hand, the mean preoperative value in the open surgery group was 4.6, and the postoperative value was 11.7.

We included in our analysis all the reported data about the status of the LHB (6 studies in total). In Table 5, we have summarized the various information found in our research and described the quality of the LHB tendon as normal (N), rupture (R), partial rupture (PR), and unstable (INS) if it was luxated/subluxated or if it showed dynamic instability. Out of 137 patients included in these 6 papers, 107 showed injuries of the LHB (78%).

In Table 5, we also reported the concomitant procedures performed in the various papers; all these procedures involved either the LHB or the acromion, and LHB tenodesis was the most commonly performed procedure.

### Complications

Table 6 shows the most important complications developed after surgery. The most common complications found in our research were rerupture, postoperative stiffness (frozen shoulder), infections, and nerve palsy. All complications involving the long head of the biceps, such as rerupture after tenodesis or postoperative ruptures, were considered separately as “minor complications.” In Table 6, we divided the arthroscopic and open surgery groups with a total of 257 shoulders assessed at the final follow-up. The total complication rate was 10.1% (26 out of 257). One study was excluded because complication data were reported on the total number of patients, and there were no specific data about either the arthroscopic or the open surgery group [32]. The arthroscopic group included 104 shoulders with a total of 10 major complications (9.7%), and only two patients needed reoperation. The open surgery group included 153 shoulders with 16 major complications (10.5%), and 5 required reoperations: 3 had a rerupture, one had an infection, and one underwent revision surgery of the LHB tenodesis for cosmetic reasons. No statistically significant difference was found between the two groups.

**Table 6:** Complications

	Patients	Rerupture		Stiffness		Infections		Nerve palsy		Minor complications (LHB)		Total	
		Total	Reoperation	Total	Reoperation	Total	Reoperation	Transient	Permanent	Total	Reoperation	Major	Total
Arthroscopy													
Lafosse et al. (2007) [23]	17	2								2		2	4
Bennett (2003) [7]	8											0	0
Novè-Josserand et al. (2016) [32]	24	5 (out of 40)											
Grueninger et al. (2014)	10											0	0
Heikenfeld et al. (2012) [20]	19	2	1	1	1					2		3	5
Novè Josserand et al. (2012) [31]	22	3										3	3
Lanz et al. (2013)	7											0	0
Bartl et al. (2011) [5]	21	1		1						1		2	3
Total	104	8	1	2	1	0		0	0	5	0	10	15
		7.7%	1%	1.9%	1%					4.8%		9.6%	14.4%
Mini-open													
Edwards et al. (2005) [13]	84	5	2	4		1	1	2		1	1	12	13
Fuchs et al. (2006) [17]	10											0	0
Kreuz et al. (2005) [22]	16			1								1	1
Novè-Josserand et al. (2016) [32]	16	5 (out of 40)											
Novè Josserand et al. (2012) [31]	13	1										1	1
Bartl et al. (2011) [5]	30	2	1									2	2
Total	153	8	3	5	0	1	1	2	0	1	1	16	17
		5.2%	2%	3.3%		0.7%	0.7%	1.3%		1.3%	1.3%	10.5%	11.1%

*Rerupture* rate of reruptures at follow-up, *Total* total number of complications, *Reoperation* number of patients that needed reoperation, *Stiffness* reduced passive range of motion, *Minor Complications (LHB)* complications involving long head of biceps were considered separately

## Discussion

The most important finding of the present study was that no statistically significant difference in clinical outcomes or complication rates was found after either arthroscopic or mini-open isolated subscapularis tendon repair, even if arthroscopic treatment led to inferior postoperative pain.

Isolated subscapularis tears are far rarer than lesions of the supraspinatus tendon or posterosuperior or anterosuperior rotator cuff tears. The literature follows this trend, and only a small number of studies have analysed isolated SSC tendon tears compared to those that have analysed anterosuperior RC tears or RC tears in general. It was suggested that RC muscles should not be evaluated as a single entity because every muscle (and tendon) has its own characteristics and functionalities.

Data after an isolated subscapular repair were systematically reviewed in this paper. In 2012, Mall et al. [25] published a similar work, but since then, no studies have been published. In the last ten years, shoulder surgery has made major advances, and arthroscopic surgery in particular is much more widespread. In our arthroscopic group, the results of 8 papers published between 2003 and 2016 were included for a total of 128 shoulders treated, while only 3 papers available at that time were considered by Mall et al. for a total of 46 shoulders. Our results confirm that SSC tendon rupture is a pathology that must be treated surgically for better clinical and functional outcomes, as shown by the improvement in the clinical and pain scores between the preoperative evaluation and the postoperative evaluation in both the arthroscopic and mini-open repair groups (Table 4). These data confirm the importance of the SSC, as it is the strongest RC muscle and the only pure intrarotator muscle of the shoulder. Our review showed very similar results in terms of clinical outcomes between the arthroscopic (postoperative CMS 84.6) and mini-open groups (postoperative CMS 82.2) and slightly better results in pain relief in patients treated with arthroscopy (mean postoperative pain score 13.3 in the arthroscopic group, 11.7 in the mini-open group). We were unable to statistically evaluate this difference for significance.

The pain generated by this particular lesion must be considered to be caused by both tendon rupture and alteration of the LHB status [39]. In our review, we reported a correlation of 78% between SSC tendon rupture and instability or lesions of the LHB. Edwards et al. suggested performing either LHB tenodesis or tenotomy, as it showed better results for pain and clinical evaluation [13], and in the research, it was found that LHB tenodesis to be the most commonly performed concomitant procedure, followed by LHB tenotomy.

Various parameters were analysed to understand their influence on the postoperative clinical outcome. Bartl et al. highlighted the negative impact of a high preoperative degree of fatty infiltration, as it is statistically correlated with positive BPT postoperatively [5]. They also suggested early operative treatment to avoid a higher degree of fatty infiltration [5]. The BPT was also correlated with preoperative tear size, as suggested by Novè-Josserand et al. in 2016 [32]. In another paper, Novè-Josserand et al. also noticed the progression of muscular fatty infiltration in the majority of patients who underwent both arthroscopy (55% of the patients showed progression of fatty infiltration) and the mini-open technique (62% showed progression). Nevertheless, they did not find any clinical implication for this anatomical aspect [31].

The most common complications reported were rerupture and stiffness in both surgical treatments. In the mini-open surgery group, Edwards et al. [13] reported the highest number of complications; in a population of 84 patients analysed, they found 5 cases of reruptures, 4 stiff shoulders, 1 infection, and 2 transient nerve palsies. Rerupture and stiffness were found with a similar incidence to that obtained by our analysis (5.95% and 4.76% reported in Edwards et al.’s paper and 5.22% and 3.27% reported in our review, respectively). This study also reported the only cases of infection and nerve palsy we found: the only patient who developed an infection had to undergo a new surgery to solve this complication, whereas the 2 cases of nerve palsy did not need any treatment and were solved spontaneously.

In the arthroscopic group, Novè-Josserrand et al. [31] reported 3 reruptures out of 22 patients, while Heikenfeld et al. [20] and Lafosse et al. [23] reported 2 reruptures each. Only two patients developed shoulder stiffness after surgical treatment in the arthroscopic surgery group. This could be explained by the reduced damage to soft tissues generated by arthroscopy compared to open surgery [4, 20].

Novè-Josserand et al. also published a paper in 2016 with a total of 40 patients treated both arthroscopically and with open surgery (24 and 16, respectively); in their population, they reported a total of 5 reruptures, but it was not specified which surgical technique had been used in each patient [32].

This review has several limitations: (1) the small amount of literature available on this rare condition means that there is relatively little collectible data; (2) the choice to include only articles published over the last 20 years led to a heterogeneity of results; (3) given the difficulty of performing the arthroscopic procedure, the results of this treatment are related to the skill and experience of the surgeon; (4) the articles have a maximum level of evidence of IV, which determines the presence of systematic bias in each study; (5) some treatments, both arthroscopic and open, were associated with concomitant procedures mainly involving the long head of the biceps (LHB) that influenced outcomes related to pre- and postoperative pain because the LHB is a major pain generator in the shoulder [8, 23, 36]; and (6) the studies included in this review reported only the average or average and range instead of the standard deviation for functional outcome measures and pain, which limits the ability to perform statistical analyses on the data to determine differences between the arthroscopic and open repair groups.

It is strongly believed that our review shows how clinical and radiological results after either arthroscopic or mini-open subscapularis tendon repair are almost equivalent. In conclusion, surgeons should always opt for the surgical technique to which they are more accustomed, and they should also treat the LHB whenever needed.

## Conclusion

There was no significant difference in clinical and functional outcomes between open and arthroscopic surgical repair of subscapularis tendon tears. However, our study demonstrated that arthroscopic repair leads to less postoperative pain. Moreover, concomitant procedures are frequently performed, and if the long head of the biceps is involved, these procedures increase pain relief in the postsurgical period.

Rerupture of the subscapularis tendon and shoulder stiffness were the most common complications observed after surgical repair. A similar percentage of these complications was found in both study groups. All the conclusions are summarized in Table 7.

**Table 7:** Capstone summary

Key finding from the review
Isolated subscapularis tears frequently occur in active patients with shoulder trauma
Clinical outcomes show excellent functional improvement and decreased pain following surgical treatment
Arthroscopic treatment is favored by the skill and experience of the surgeon and shows better results in postoperative pain management than open treatment
Treatment of concomitant diseases results in improved clinical outcome

## Data Availability

The datasets used and analyzed during the current study are available from the corresponding author.
